# Anatomy of a Fever

**DOI:** 10.1371/journal.pbio.0040305

**Published:** 2006-08-22

**Authors:** Liza Gross

Many parents experience fear and anxiety when their child comes down with a fever, unaware that fever is an ancient, often beneficial, response to infection. The fever response is conserved across all mammals and many vertebrate classes. (Even reptiles and other cold-blooded animals fare better against infection when they develop fever by soaking up the sun's heat.) Among other potential adaptive benefits, a higher temperature can inhibit the growth of bacterial strains that lack sophisticated mechanisms for coping with heat shock.

Fever, which is mediated by a lipid called prostaglandin E_2_ (PGE_2_), can pass through multiple temperature phases. While it's well established that PGE_2_ originating in brain cells causes the second and later phases, the initial phase of fever has proven difficult to characterize. Of particular interest is whether fever onset is triggered by PGE_2_ that originates inside or outside the brain a question that has dogged researchers for nearly three decades. Now, Alexandre Steiner, Andrej Romanovsky, and colleagues provide evidence that PGE_2_ synthesis doesn't begin in the brain as previously thought, but in the lungs and liver. They also describe the molecular mechanisms that produce PGE_2_ in these organs.

Many of the mechanisms of fever have been established by exposing rodents to bacterial endotoxins called lipopolysaccharides (LPS). The first phase of LPS-induced fever starts within 30 minutes after exposure to the pyrogen. During the second and subsequent phases between 90 minutes and 12 hours after LPS administration brain cells increase production (called upregulation) of enzymes involved in PGE_2_ synthesis. Thus, fever starts about an hour before the PGE_2_-synthesizing enzymes cyclooxygenase-2 (COX-2) and microsomal PGE synthase-1 (mPGES-1) are upregulated in the brain, suggesting that fever must be triggered by PGE_2_ produced peripherally, outside the brain.

To test this hypothesis, Steiner et al. gave rats an intravenous (IV) solution of PGE_2_ bound to albumin, the primary transporter of PGE_2_ in the blood. Controls received an IV albumin solution. After confirming that the PGE_2_ infusion induced fever, the researchers collected venous and arterial blood from LPS-treated rats. (PGE_2_ synthesized in tissues amasses in venous blood; arterial blood delivers PGE_2_ to the brain.) At the onset of fever, PGE_2_ was significantly elevated both in the venous and arterial blood.

To investigate the origin of fever-inducing PGE_2_, Steiner et al. used an antibody-based technique. (Antibodies are too large to cross the blood–brain barrier.) Rats were pretreated with IV anti-PGE_2_ antibody serum (controls received normal serum) and then injected with IV LPS. The first phase of LPS fever was significantly attenuated by the antibody (but not the serum), which was found in the blood but not in the brain. These results show that LPS-induced fever is triggered by circulating PGE_2_.

**Figure:**
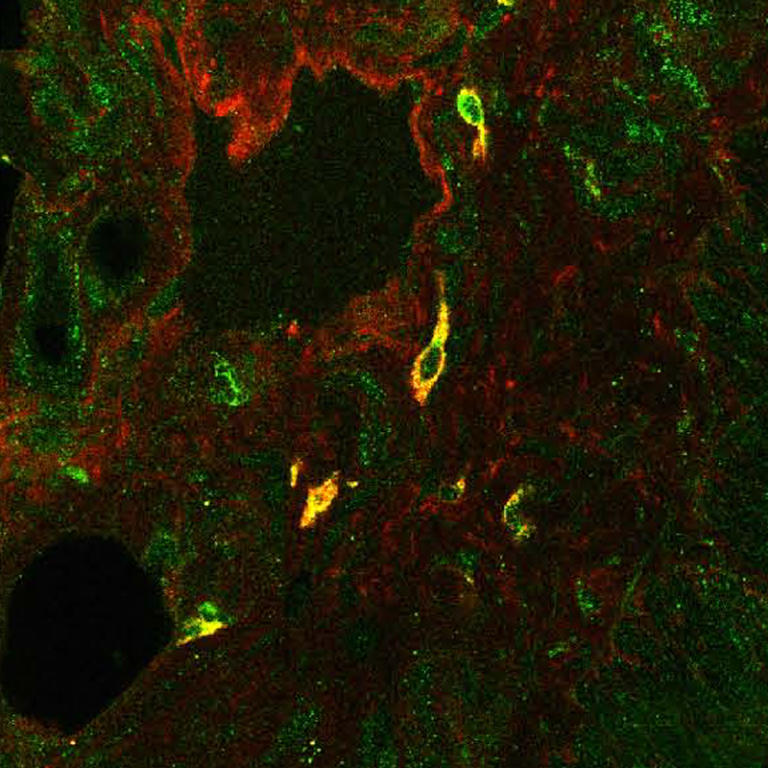
Cyclooxygenase-2 (green immunofluorescence) and the macrophage marker ED2 (red immunofluorescence) in rat lung at the onset of bacterial lipopolysaccharide-induced fever (photo: Jordi Serrats).

A previous study from the Romanovsky laboratory reported that onset of fever is accompanied by significantly increased transcription of COX-2 and mPGES-1 in the lung and liver, and a moderate increase of the COX-2 transcript in the hypothalamus (the body's “fever center”). In this study, they examined the protein distribution of these enzymes in all three tissues. After LPS exposure, the content of neither protein was increased in the hypothalamus. But the lung and liver did show increased COX-2 expression primarily in macrophages, which play a role in the inflammatory response along with activation of an enzyme (cytosolic phospholipase A2) involved in the early steps of PGE_2_ synthesis. These organs, but not the hypothalamus, also showed signs of inflammatory signaling. Interestingly, the researchers did not find increased levels of mPGES-1, which facilitates the final step of PGE_2_ synthesis, suggesting that the cell's normal supply of this enzyme manages the task.

Altogether, these results provide a cellular and molecular portrait of the first phase of fever and show that it depends, at least in part, on PGE_2_ that originates in peripheral tissues.

